# Assessment of depression, anxiety and stress levels in the Ecuadorian general population during social isolation due to the COVID-19 outbreak: a cross-sectional study

**DOI:** 10.1186/s12888-021-03214-1

**Published:** 2021-04-28

**Authors:** Hans Mautong, Jorge Andrés Gallardo-Rumbea, Geovanny Efraín Alvarado-Villa, Juan Carlos Fernández-Cadena, Derly Andrade-Molina, Carlos Enrique Orellana-Román, Iván Cherrez-Ojeda

**Affiliations:** 1 School of Medicine, Universidad de Especialidades Espíritu Santo, Samborondon, Ecuador; 2 Laboratory of Omic Sciences, School of Medicine, Universidad de Especialidades Espíritu Santo, Samborondón, Ecuador; 3 Neurosciences Institute, Guayaquil, Ecuador; 4 Respiralab, Respiralab Research Group, Guayaquil, Ecuador

**Keywords:** Coronavirus, COVID-19, Social isolation, Depression, Anxiety, Stress, Mental health

## Abstract

**Background:**

Coronavirus disease 2019 (COVID-19) has become a global pandemic with serious consequences that have led to the implementation of unprecedented social isolation measures. At the early stages of the pandemic, Ecuador was one of the most affected countries in Latin America. The objective of this study was to assess the levels of depression, anxiety and stress in the Ecuadorian general population during the social isolation period due to COVID-19.

**Methods:**

A web-based survey consisting of 31 short-answer and multiple-choice questions was administered to the general population from April 22–May 3, 2020. Mental health status was assessed through the Depression, Anxiety and Stress Scale-21 Items (DASS-21) questionnaire. Ordinal logistic analyses were used to identify potential risk factors associated with the severity of mental health issues.

**Results:**

A total of 626 individuals were included. Most of them were females (60.5%), and their mean age was 29.6 ± 11.7 years. Approximately 17.7% of the respondents had moderate to very severe levels of depression, 30.7% had similar levels of anxiety, and 14.2% experienced stress. Female sex, younger age, student status, and having a relative diagnosed with COVID-19 were associated with significantly higher levels of depression, anxiety and stress. Ordinal regression models showed that being a student was a risk factor for having more severe levels of depression (OR = 3.67; 95% CI = 2.56–5.26, *p:* 0.0001), anxiety (OR= 1.86; 95% CI= 1.35–2.55, *p:* 0.0001), and stress (OR = 2.17; 95% CI= 1.47–3.19, *p:* 0.0001). Having a relative with COVID-19 was also found to be a risk factor only for depression (OR= 1.70; 95% CI= 1.03–2.80, *p:* 0.036) and anxiety (OR = 2.17; 95% CI= 1.35–3.47, *p:* 0.001). Additionally, male sex,  older age, and having more children were found to be protective factors for the three conditions.

**Conclusions:**

Our findings suggest that social isolation due to the COVID-19 outbreak has impacted the mental health of the general population in Ecuador. We identified potential risk and protective factors that could serve as a foundation from which to develop psychological strategies to safeguard the mental health of our population during the current pandemic.

**Supplementary Information:**

The online version contains supplementary material available at 10.1186/s12888-021-03214-1.

## Background

Coronavirus disease 2019 (COVID-19) is a novel infectious disease caused by severe acute respiratory syndrome coronavirus 2 (SARS-CoV-2) [1]. Due to its rapid global spread, the disease rapidly escalated to the category of a global pandemic, as declared by the World Health Organization (WHO) on March 11, 2020 [2]. To prevent disease transmission, individuals who might have been exposed to COVID-19 are quarantined to keep them separated from others. Quarantine also helps prevent the spread of disease by people who may not know they are sick during the presymptomatic incubation period and by people who remain asymptomatic throughout the clinical course.

The first case of COVID-19 in Ecuador occurred on February 29, 2020. A few weeks later, Ecuador’s largest city, Guayaquil, reported approximately 2200 cases, which corresponded to 70% of the cases in the country [3]. An estimate of 6700 people died in Guayaquil and Guayas province within the first 2 weeks of April. This death toll was more than 6 times greater than the usual death toll of previous years (usually approximately 1,000 deaths) during the same period of time [4]. Hospitals were rapidly saturated, and even funeral workers were unable or unwilling to collect the bodies due to excessive death cases. As the pandemic continued to deepen in poorly prepared developing countries, the situation in Ecuador became devastating. For this reason, the government adopted social isolation measures with strict mobility restrictions and the suspension of all nonessential face-to-face entertainment, academic, and work activities; however, the shocking number of deaths has earned Ecuador the title of “the emerging epicenter of COVID-19 in Latin America” from the international media [3, 4].

Recent evidence has suggested that previous epidemics (due to Ebola virus [5] and other coronaviruses [6, 7]) have had profound effects on the mental health of individuals, resulting in anxiety, depression and even posttraumatic stress disorder (PTSD) in different populations; there is growing evidence that the same is true for the current COVID-19 pandemic [8–11]. The strict lockdown and social isolation regulations implemented in Ecuador have certainly disrupted the daily lives of many Ecuadorians. Moreover, the lack of knowledge about COVID-19, misinformation from the media, the lack of effective treatments, travel restrictions, significant economic losses, strict isolation requirements, and more importantly, the alarming mortality rate [12, 13] may result in negative psychological consequences (such as higher levels of depression, anxiety and stress) during the COVID-19 pandemic in Ecuador. There are few data available regarding this issue in Latin America [14, 15]. To address this knowledge gap, we assessed the mental health of the Ecuadorian general population and the associated risk factors during social isolation due to COVID-19.

## Methods

### Study design and population

This cross-sectional study used a web-based survey made on QuestionPro® consisting of 31 short-answer and multiple-choice questions to collect data from Ecuador’s general population. Due to limited time and resources, we adopted the snowball sampling method. The survey was broadcast through social media channels to avoid face-to-face interactions and facilitate access to socially isolated people. A brief introduction of the objective of the study alongside with the URL link to the survey was sent to potential participants, which were encouraged to forward it to their own social media contacts. Once they opened the link, they were redirected to the QuestionPro® webpage, where they could access the survey. From April 22 (38 days after the obligatory social isolation period was announced) to May 3, 2020 (the day social isolation measures were lifted), Ecuadorians anonymously answered the questionnaire, and participants were informed of the purpose of the study and enrolled in it. The survey took approximately 12 min to complete. To encourage survey completion, a local supermarket gift card was offered to participants in a raffle. The survey consisted of three modules that collected information on 1) demographics and the Depression, Anxiety and Stress Scale-21 Items (DASS-21); 2) exposure to COVID-19 and disruptions to daily life activities; and 3) general health status.

The only inclusion criterion was that the participants had to be over 18 years old. Exclusion criteria included participants who were outside of Ecuador during the fulfillment of the survey and those who did not fill out the survey consciously. The participant’s location was addressed with a feature from QuestionPro®, which indicated the approximate location of the participant. Similarly, a pair of test questions were added to the survey, allowing us to identify which surveys were answered consciously and which were not. The test question consisted in choosing the answer dictated by the slogan. If the participant did not select the dictated answer, their response was considered biased. In case of being located outside Ecuador, or if any of the test questions were answered incorrectly, the respondent was excluded from the study. We used the IP control tool from QuestionPro®, which allowed us to remove potential repeated surveys, however no survey repetition was found. A total of 780 individuals completed the questionnaire (response rate = 59.23%), 154 were excluded from the study according to exclusion criteria, and 626 participants were included in the final analysis.

### Data collection

The first module in the survey (Module A) consisted of two parts: the demographic information of participants (sex, pregnancy status, age, marital status, level of education, location, area of residency, type of residence, number of children, cohabitation with a person with physical and/or intellectual disabilities, employment/educational situation, socioeconomic status, chronic diseases, medications, depressive disorder diagnoses, and neighborhood) and the questions from the DASS-21. The validated Spanish version of the DASS-21 was used to evaluate the mental health status of socially isolated Ecuadorians during the COVID-19 outbreak; this questionnaire was validated by Daza, P. in 2002 [16]. The DASS-21 is composed of 21 items divided into three subcategories (depression, anxiety and stress), which are each measured on a 4-point (0–3) Likert-type scale (ranging from “did not apply to me at all” = 0 to “applied to me very much or most of the time” = 3). At the beginning, participants were asked how much over the past month the statements in DASS-21 applied to them. Then, the depression subscale addressed dysphoria, hopelessness, devaluation of life, self-deprecation, lack of interest/involvement, anhedonia and inertia. The anxiety subscale addressed autonomic arousal, skeletal muscle effects, situational anxiety, and the subjective experience of anxious affect. Last, the stress subscale, which is sensitive to levels of chronic nonspecific arousal, addressed difficulty relaxing, nervous arousal, and being easily upset/agitated, irritable/overreactive and impatient. Scores for depression, anxiety and stress were calculated by summing the scores for the relevant items. Scores were obtained by summing the individual items for each scale and multiplying them by 2. The subscale scores ranged from 0 to 42, and the total scores ranged from 0 to 126. A score greater than 9 on the depression subscale, 7 on the anxiety subscale, and 14 on the stress subscale indicated the presence of those conditions [17]. Additionally, the DASS-21 questionnaire was included in the demographics section to prevent careless answers due to the length of the survey.

Next, questions about exposure and daily life disruptions due to COVID-19 were asked in module B to assess the participants’ perception of the pandemic during the social isolation period. This module included questions about which aspects of their daily lives had been affected by the pandemic and what measures they were personally taking to avoid contracting COVID-19. Module C consisted of questions about their general physical/mental health and information about drug consumption and disabilities.

### Statistical analysis

All statistical analyses were performed using SPSS for Windows (version 23.0; SPSS Inc., Chicago, Illinois). The Kolmogorov-Smirnov test was used to test for the normality of distribution. Categorical variables are presented as percentages. Continuous variables are presented as the means ± SDs or medians (interquartile ranges). The Mann-Whitney U test and one-way ANOVA were used to compare variables with two or more categories, respectively. Correlations were determined with Spearman correlation coefficients. Ordinal logistic analysis was used to determine potential associations between independent variables and dependent variables. Only variables that showed statistical significance in univariate ordinal regression analyses were included in the multivariate model. Statistical significance was defined by a 2-tailed *p* < 0.05.

### Ethical considerations

Before answering the survey, participants were informed in detail about the purpose of the study, and all participants provided electronic informed consent. This study was conducted in accordance with the Declaration of Helsinki and was approved by the Expedited Ethics Committee of the Ecuadorian Health Ministry (Approval N° 024–2020). With the information recollected in the survey, personal identification was not possible; as such, anonymity and personal data protection were preserved.

## Results

### Sociodemographic characteristics of the study population

The baseline sociodemographic characteristics of the 626 participants are shown in Table 1. The  mean age of the participants was 29.6 ± 11.7 years, and the majority of them were female (60.5%). Approximately half of the participants (50.3%) were students, and 60.7% were single. Most of the respondents (64.7%) lived in Guayas Province, and as much as 40.4% lived in its capital, Guayaquil. Approximately 16.9% of participants experienced COVID-19 symptoms, 10.7% had a relative diagnosed with COVID-19, and just 5.6% of them had a confirmed COVID-19 diagnosis.

**Table 1:** Baseline sociodemographic characteristics

Variables	*n* = 626
Age, years (Mean ± SD)	29.6 ± 11.7
Sex	
Male	247 (39.5%)
Female	379 (60.5%)
Relationship status	
Single	420 (67.1%)
Married	147 (23.5%)
Widow(er)	2 (0.3%)
Divorced/separated	38 (6.1%)
Free union	19 (3.0%)
Level of education	
Primary education	2 (0.3%)
Secondary education	253 (40.4%)
Technical	23 (3.7%)
Bachelor or university	268 (42.8%)
Postgraduate	80 (12.8%)
Location	
Guayas	405 (64.7%)
Guayaquil	253 (40.4%)
Samborondon	104 (16.6%)
Daule	30 (4.8%)
Other cities in Guayas	18 (2.9%)
Rest of the country	221 (35.3%)
Type of residence	
Own apartment	74 (11.8%)
Rented apartment	50 (8.0%)
Own house	381 (60.9%)
Rented house	30 (4.8%)
Family home	90 (14.4%)
Other	1 (0.2%)
Student	315 (50.3%)
Number of children (Mean ± SD)	0.67 ± 1153
Number of people living with respondent (Mean ± SD)	4.48 ± 1785
Cohabitants with physical and/or intellectual disabilities.	68 (10.9%)
COVID-19 symptoms	106 (16.9%)
Confirmed COVID-19 diagnosis	35 (5.6%)
Relative diagnosed with COVID-19	67 (10.7%)
Cigarette consumption	
None	537 (85.9%)
Less than before	72 (11.5%)
As before	8 (1.3%)
More than before	8 (1.3%)
Alcohol consumption	
None	356 (57.2%)
Less than before	242 (38.9%)
As before	19 (3.1%)
More than before	5 (0.8%)

*Abbreviations*: *n* number, *SD* standard deviation, *COVID-19* coronavirus disease 2019

### Levels of depression, anxiety, and stress

The median depression score was 6 (IQR 2–10). Approximately 17.7% of participants reported moderate to very severe levels of depression. The median anxiety score was 6 (IQR 2–10). Interestingly, 30.7% of the respondents reported moderate to very severe anxiety levels. The median stress score was 10 (IQR 5–14), which was higher than the scores for anxiety and depression; however, the proportion of participants with moderate to very severe levels of stress was 14.2% (Table 2).

**Table 2:** Levels of depression, anxiety and stress

Variable	Severity	n (%)	Median (IQR)
Depression	Normal	429 (68.6%)	6 (2–10)
	Mild	85 (13.6%)	
	Moderate	73 (11.7%)	
	Severe	19 (3.0%)	
	Very severe	19 (3.0%)	
Anxiety	Normal	377 (60.3%)	6 (2–10)
	Mild	56 (9.0%)	
	Moderate	118 (18.9%)	
	Severe	35 (5.6%)	
	Very severe	39 (6.2%)	
Stress	Normal	482 (77.2%)	10 (5–14)
	Mild	53 (8.5%)	
	Moderate	52 (8.3%)	
	Severe	32 (5.1%)	
	Very severe	5 (0.8%)	

### Associations of sociodemographic characteristics with depression, anxiety, and stress levels

**Fig. 1:**
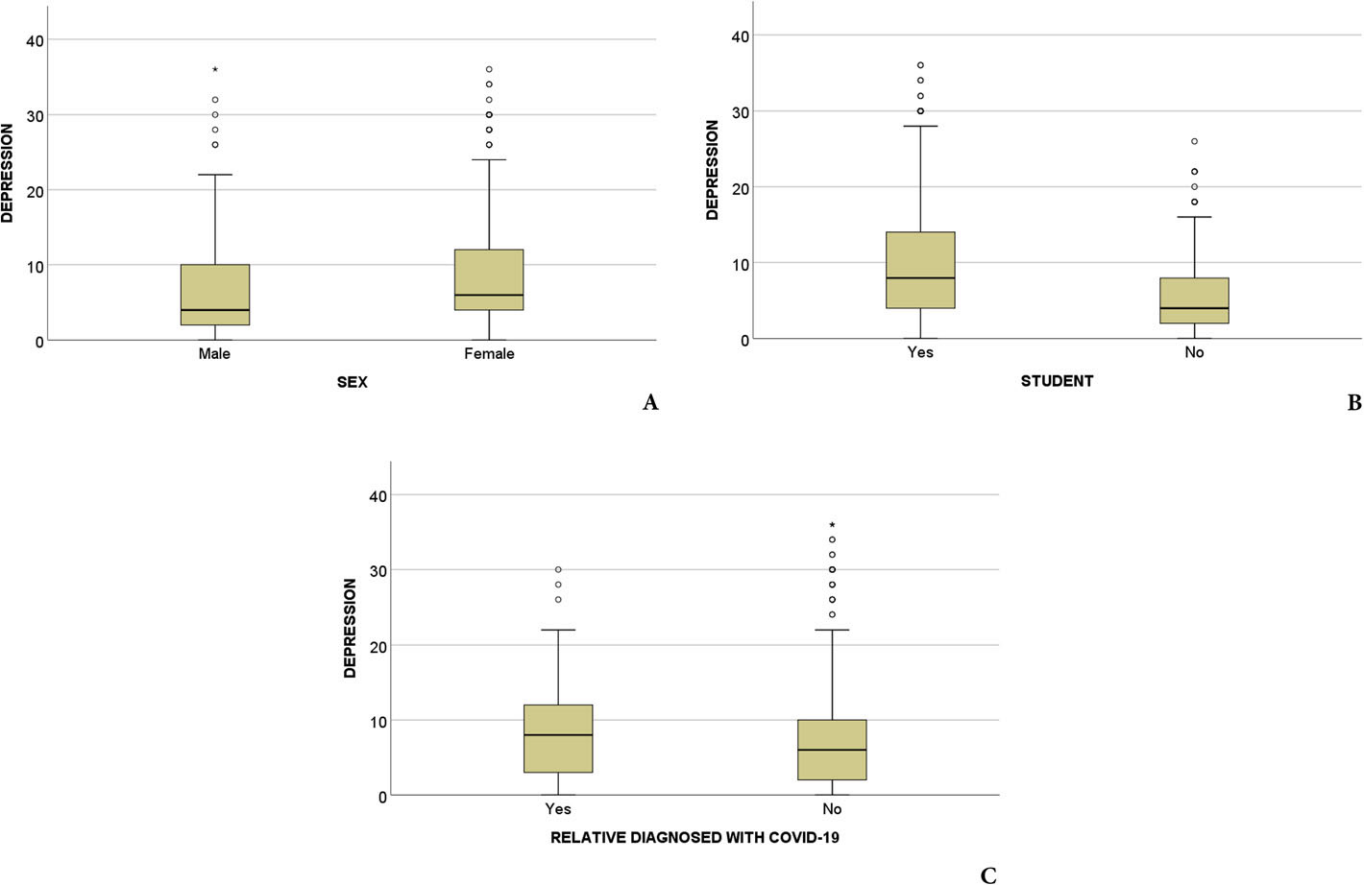
Comparison of the levels of depression according to sex (**a**), student status (**b**), and having a relative diagnosed with COVID-19 (**c**)

As shown in Table 3, the level of depression among females was significantly higher than that among males (*p* < 0.0001) (Fig. 1a). The level of depression was higher among single individuals than that among those with other relationship statuses (*p* < 0.0001). The level of depression was significantly higher among high school graduates than among the other groups (*p* < 0.0001). Students tended to have significantly higher levels of depression than nonstudents (*P* < 0.0001) (Fig. 1b). Finally, respondents who had a relative diagnosed with COVID-19 had significantly higher levels of depression than those who did not (*p* = 0.032) (Fig. 1c).

**Table 3:** Association of sociodemographic characteristics with depression levels

		Depression				
Variable	Rate	Severity Frequency (percentage)				
		Normal	Mild	Moderate	Severe	Extremely severe
**Sex**						
Male	4 (2–10)	183 (42.7)	26 (30.6)	28 (38.4)	6 (31.6)	4 (21.1)
Female	6 (4–12)	246 (57.3)	59 (69.4)	45 (61.6)	13 (68.4)	15 (78.9)
*P*	0.000					
**Relationship Status**						
Single	8 (4–12)	250 (58.3)	70 (82.4)	65 (89)	16 (84.2)	19 (100)
Married	4 (2–6)	132 (30.8)	6 (7.1)	6 (8.2)	3 (15.8)	0 (0)
Widow(er)	3 (2–4)	2(0.5)	0 (0)	0 (0)	0 (0)	0 (0)
Divorced/separated	4 (2–6)	32(7.5)	4 (4.7)	1 (1.4)	0 (0)	0 (0)
Free union	4 (2–10)	13(3.0)	5 (5.9)	1 (1.4)	0 (0)	0 (0)
*P*	0.000					
**Level of education**						
None	0 (0–0)	0(0.0)	0 (0)	0 (0)	0 (0)	0 (0)
Primary education	1 (0–2)	2 (0.5)	0 (0)	0 (0)	0 (0)	0 (0)
Secondary education	8 (4–14)	140 (32.6)	45 (52.9)	43 (58.9)	12 (63.2)	13 (68.4)
Technical	4 (2–10)	17 (4)	2 (2.4)	2 (2.7)	1 (5.3)	1 (5.3)
University	6 (2–9)	201 (46.9)	33 (38.8)	25 (34.2)	4 (21.1)	5 (26.3)
Postgraduate	4 (0–8)	69 (16.1)	5 (5.9)	3 (4.1)	2 (10.5)	0 (0)
*P*	0.000					
**Location**						
Guayas	6 (2–10)	274 (63.9)	57 (67.1)	46 (63)	15 (78.9)	13 (68.4)
Rest of the country	6 (2–10)	155 (36.1)	28 (32.9)	27 (37)	4 (21.1)	6 (31. 6)
*P*	0.491					
**Type of residence**						
Own apartment	6 (2–12)	51 (11.9)	11 (12.9)	6 (8.2)	1 (5.3)	4 (21.1)
Rented apartment	6 (2–12)	33 (7.7)	6 (7.1)	6 (8.2)	4 (21.1)	1 (5.3)
Own house	6 (2–10)	265 (61.8)	55 (64.7)	41 (56.2)	9 (47.1)	11 (57.9)
Rented house	6 (4–14)	20 (4.7)	1 (1.2)	8 (11)	1 (5.3)	0 (0)
Family home	6 (2–12)	59 (13.8)	12 (14.3)	12 (16.2)	4 (21.1)	3 (15.8)
Other	2 (2–2)	1 (0.2)	0 (0)	0 (0)	0 (0)	0 (0)
*P*	0.757					
**Cohabitants with physical and/or intellectual disabilities**						
Yes	6 (2–10)	44 (10.3)	12 (14.3)	6 (8.2)	4 (21.1)	1 (5.3)
No	6 (2–10)	385 (89.7)	72 (85.7)	67 (91.8)	15 (78.9)	18 (94.7)
*P*	0.494					
**Student**						
Yes	8 (4–14)	175 (40.8)	50 (58.8)	54 (75)	14 (73.7)	19 (100)
No	4 (2–8)	254 (59.2)	35 (41.2)	18 (25)	5 (26.3)	0 (0)
*P*	0.000					
**COVID-19 symptoms**						
Yes	6 (2–12)	67 (15.6)	16 (18.8)	15 (20.5)	4 (21.1)	4 (21.1)
No	6 (2–10)	362 (84.4)	69 (81.2)	58 (79.5)	15 (78.9)	15 (78.9)
*P*	0.826					
**COVID-19 diagnosis**						
Yes	6 (2–10)	24 (5.6)	7 (8.2)	1 (1.4)	1 (5.3)	2 (10.5)
No	6 (2–10)	405 (94.4)	78 (91.8)	72 (98.6)	18 (94.7)	17 (89.5)
*P*	0.455					
**Relative diagnosed with COVID-19**						
Yes	8 (2–12)	38 (8.9)	13 (15.3)	10 (13.7)	4 (21.1)	2 (10.5)
No	6 (2–10)	391 (91.1)	72 (84.7)	63 (86.3)	15 (78.9)	17 (89.5)
*P*	0.032

**Fig. 2:**
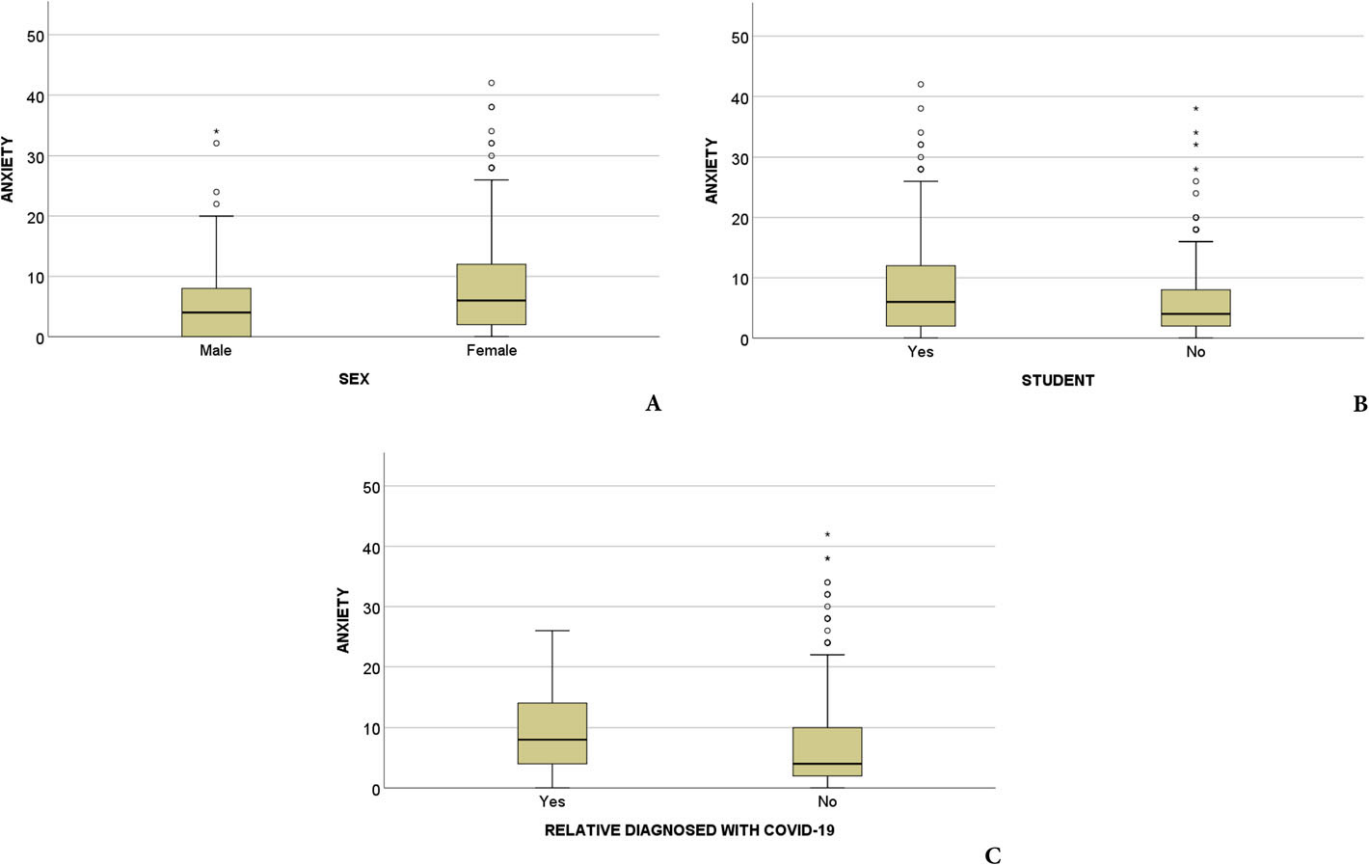
Comparison of the levels of anxiety according to sex (**a**), student status (**b**), and having a relative diagnosed with COVID-19 (**c**)

Concerning anxiety levels (Table 4), females had significantly higher anxiety levels than males (*p* = 0.0001) (Fig. 2a). The level of anxiety was higher among single individuals than among the other groups (*p* = 0.0001). In terms of the level of education, the level of anxiety was significantly higher among high school graduates than among the other groups (*p* = 0.0001). Students tended to have significantly higher levels of anxiety than nonstudents (*p*: 0.0001) (Fig. 2b). Respondents who had COVID-19 symptoms (*p* = 0.002) or a relative diagnosed with COVID-19 (*p* = 0.004) had significantly higher levels of anxiety than those who did not (Fig. 2c).

**Table 4:** Association of sociodemographic characteristics with anxiety levels

		Anxiety				
Variable	Rate	Severity Frequency (percentage)				
		Normal	Mild	Moderate	Severe	Extremely severe
**Sex**						
Male	4 (0–8)	173 (45.9)	21 (37.5)	36 (30.5)	12 (34.3)	5 (12.8)
Female	6(2–12)	204 (54.1)	35 (62.5)	82 (69.5)	23 (65.7)	34 (87.2)
*P*	0.000					
**Relationship status**						
Single	6(2–12)	236 (62.6)	38 (67.9)	82 (69.5)	29 (82.9)	35 (89.7)
Married	4(0–8)	104 (27.6)	14 (25)	21 (17.8)	4 (11.4)	4 (10.3)
Widow(er)	0 (0–0)	2 (0.5)	0 (0)	0 (0)	0 (0)	0 (0)
Divorced/separated	6 (2–10)	23 (6.1)	2 (3.6)	10 (8.5)	2 (5.7)	0 (0)
Free union	6 (2–10)	12 (3.2)	2 (3.6)	5 (4.1)	0 (0)	0 (0)
*P*	0.0001					
**Level of education**						
None	0 (0–0)	0 (0)	0 (0)	0 (0)	0 (0)	0 (0)
Primary education	2 (0–4)	2 (0.5)	0 (0)	0 (0)	0 (0)	0 (0)
Secondary education	6 (2–10)	136 (36.1)	18 (32.1)	54 (45.8)	20 (57.1)	25 (64.1)
Technical	4 (0–10)	16 (4.2)	1 (1.8)	3 (2.5)	1 (2.9)	2 (5.1)
University	4 (2–10)	166 (44)	29 (51.8)	50 (42.4)	14 (40)	9 (23.1)
Postgraduate	4 (0–8)	57 (15.1)	8 (14.3)	11 (9.3)	0 (0)	3 (7.7)
*P*	0.004					
**Location**						
Guayas	6 (2–10)	241 (63.9)	40 (71.4)	77 (65.3)	19 (54.3)	28 (71.8)
Rest of the country	4 (2–10)	136 (36.1)	16 (28.6)	41 (34.7)	16 (45.7)	11 (28.2)
*P*	0.432					
**Type of residence**						
Own apartment	6 (2–10)	41 (10.9)	10 (17.9)	12 (10.2)	4 (11.4)	6 (15.4)
Rented apartment	6 (2–10)	28 (7.4)	6 (10.7)	8 (6.8)	2 (5.7)	6 (15.4)
Own house	6 (2–10)	235 (62.3)	31 (55.4)	78 (66.1)	19 (54.3)	18 (46.2)
Rented house	4 (0–10)	21 (5.6)	1 (1.8)	5 (4.2)	3 (8.6)	0 (0)
Family home	6 (2–12)	51 (13.5)	8 (14.3)	15 (12.7)	7 (20)	9 (23.1)
Other	4 (4–4)	1 (0.3)	0 (0)	0 (0)	0 (0)	0 (0)
*P*	0.619					
**Cohabitants with physical and/or intellectual disabilities**						
Yes	6 (4–10)	38 (10.1)	10 (17.9)	13 (11.1)	2 (5.7)	4 (10.3)
No	4 (2–10)	339 (89.9)	46 (82.1)	104 (88.9)	33 (94.3)	35 (89.7)
*P*	0.135					
**Student**						
Yes	6 (2–12)	169 (44.8)	24 (42.9)	68 (57.6)	21 (61.8)	30 (76.9)
No	4 (2–8)	208 (55.2)	32 (57.1)	50 (42.4)	13 (38.2)	9 (23.1)
*P*	0.001					
**COVID-19 symptoms**						
Yes	8 (2–14)	45 (11.9)	15 (26.8)	24 (20.3)	11 (31.4)	11 (28.2)
No	4 (2–10)	332 (88.1)	41 (73.2)	94 (79.7)	24 (68.6)	28 (71.8)
*P*	0.002					
**COVID-19 diagnosis**						
Yes	8 (2–14)	16 (4.2)	5 (8.9)	6 (5.1)	5 (14.3)	3 (7.7)
No	6 (2–10)	361 (95.8)	51 (91.1)	112 (94.9)	30 (85.7)	36 (92.3)
*P*	0.164					
**Relative diagnosed with COVID-19**						
Yes	8 (4–14)	27 (7.2)	10 (17.9)	17 (14.4)	7 (20)	6 (15.4)
No	4 (2–10)	350 (92.8)	46 (82.1)	101 (85.6)	28 (80)	33 (84.6)
*P*	0.004

**Fig. 3:**
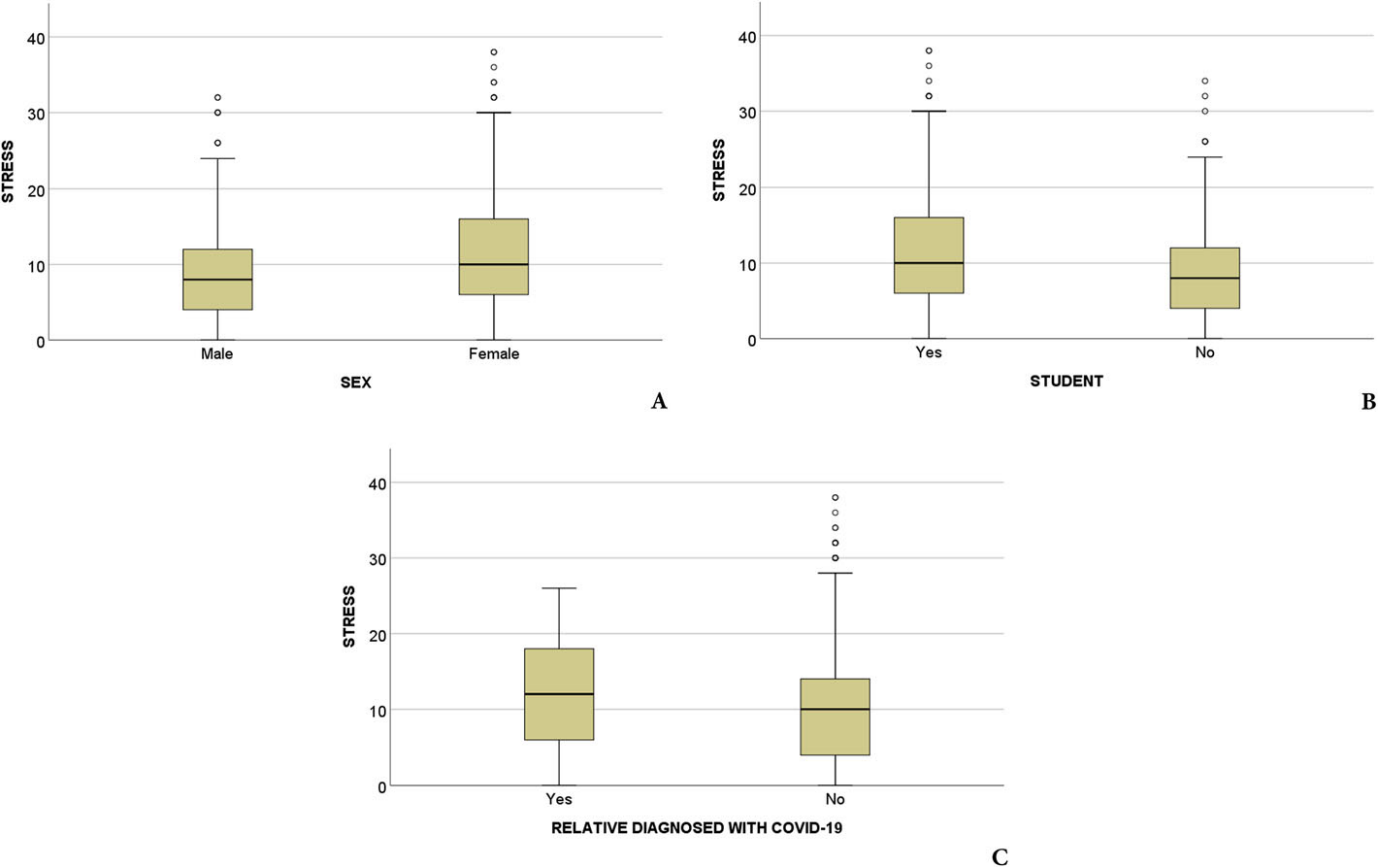
Comparison of the levels of stress according to sex (**a**), student status (**b**), and having a relative diagnosed with COVID-19 (**c**)

With regard to stress levels, as shown in Table 5, females had significantly higher levels than males (*p* = 0.0001) (Fig. 3a). The level of stress was higher among single individuals than among the other groups (*p* = 0.0001). The level of stress was significantly higher among those with secondary education degrees than among the other groups (*p* = 0.0001). Students tended to have significantly higher levels of stress than nonstudents (p: 0.0001) (Fig. 3b). Finally, respondents who had a relative diagnosed with COVID-19 had significantly higher levels of stress than those who did not (p: 0.032) (Fig. 3c).

**Table 5:** Association of sociodemographic characteristics with stress levels

		Stress				
Variable	Rate	Severity Frequency (percentage)				
		Normal	Mild	Moderate	Severe	Extremely severe
**Sex**						
Male	8 (4–12)	213 (44.2)	13 (24.5)	13 (25)	8 (25)	0 (0)
Female	10 (6–16)	269 (55.8)	40 (75.5)	39 (75)	24 (75)	5 (100)
*P*	0.000					
**Relationship status**						
Single	10 (6–16)	306 (63.5)	39 (73.6)	42 (80.2)	28 (87.5)	4 (80)
Married	8 (4–12)	128 (26.6)	7 (13.2)	7 (13.5)	4 (12.5)	1 (20)
Widow(er)	6 (6–6)	2 (0.4)	0 (0)	0 (0)	0 (0)	0 (0)
Divorced/separated	8 (2–14)	29 (6)	5 (9.4)	3 (5.8)	0 (0)	0 (0)
Free union	8 (6–14)	17 (3.5)	2 (3.8)	0 (0)	0 (0)	0 (0)
*P*	0.000					
**Level of education**						
None	0 (0–0)	0 (0)	0 (0)	0 (0)	0 (0)	0 (0)
Primary education	7 (6–8)	2 (0.4)	0 (0)	0 (0)	0 (0)	0 (0)
Secondary education	12 (6–18)	173 (35.9)	31 (58.5)	25 (48.1)	21 (65.6)	3 (60)
Technical	8 (6–18)	17 (3.5)	1 (1.9)	2 (3.8)	2 (6.3)	1 (20)
University	8 (4–14)	219 (45.4)	19 (35.8)	21 (40.4)	8 (25)	0 (0)
Postgraduate	8 (4–14)	71 (14.7)	2 (3.8)	4 (7.7)	1 (3.1)	1 (20)
*P*	0.000					
**Location**						
Guayas	10 (4–14)	311 (64.5)	29 (54.7)	40 (76.9)	20 (62.5)	4 (80)
Rest of the country	10 (6–14)	171 (35.5)	24 (45.3)	12 (23.1)	12 (37.5)	1 (20)
*P*	0.851					
**Type of residence**						
Own department	10 (6–16)	54 (11.2)	7 (13.2)	4 (7.7)	7 (21.9)	1 (20)
Rented apartment	10 (6–14)	39 (8.1)	4 (7.5)	3 (5.8)	3 (9.4)	1 (20)
Own house	8 (4–14)	303 (62.9)	29 (54.7)	32 (61.5)	14 (43.8)	3 (60)
Rented house	10 (4–14)	25 (5.2)	2 (3.8)	2 (3.8)	1 (3.1)	0 (0)
Family home	10 (6–16)	60 (12.4)	11 (20.8)	11 (21.2)	7 (21.9)	0 (0)
Other	0 (0–0)	1 (0.2)	0 (0)	0 (0)	0 (0)	0 (0)
*P*	0.619					
**Cohabitants with physical and/or intellectual disabilities**						
Yes	10 (6–16)	49 (10.2)	6 (11.3)	5 (9.6)	6 (18.8)	0 (0)
No	10 (4–14)	432 (89.8)	47 (88.7)	47 (90.4)	26 (81.3)	5 (100)
*P*	0.402					
**Student**						
Yes	10 (6–16)	221 (45.9)	31 (58.5)	28 (54.9)	27 (84.4)	4 (80)
No	8 (4–12)	261 (54.1)	22 (41.5)	23 (45.1)	5 (15.6)	1 (20)
*P*	0.000					
**COVID-19 symptoms**						
Yes	11 (6–16)	74 (15.4)	15 (28.3)	11 (21.2)	5 (15.6)	1 (20)
No	10 (4–14)	408 (84.6)	38 (71.7)	41 (78.8)	27 (84.4)	4 (80)
*P*	0.054					
**COVID-19 diagnosis**						
Yes	8 (4–18)	25 (5.2)	3 (5.7)	6 (11.5)	1 (3.1)	0 (0)
No	10 (6–14)	457 (94.8)	50 (94.3)	46 (88.5)	31 (96.9)	5 (100)
*P*	0.939					
**Relative diagnosed with COVID-19**						
Yes	12 (6–18)	45 (9.3)	9 (17)	8 (15.4)	5 (15.6)	0 (0)
No	10 (4–14)	437 (90.7)	44 (83)	44 (84.6)	27 (84.4)	5 (100)
*P*	0.032

Surprisingly, having COVID-19 symptoms was not associated with significantly higher levels of depression or stress. In addition, having a positive COVID-19 diagnosis was not associated with significantly higher levels of depression, anxiety or depression.

### Correlations of mental and physical health perceptions with depression, anxiety, and stress levels

As shown in Table 6, the levels of depression, anxiety and stress had significant inverse correlations with age, the number of children, the subjective perception of overall health, and the subjective perception of mental health. On the other hand, a participant’s level of concern with contracting COVID-19 had significant direct correlations with the levels of depression (*r* = 0.121; *p* < 0.002), anxiety (*r* = 0.244; *p* < 0.0001), and stress (*r* = 0.244; *p* < 0.05). In contrast, the number of people living with the respondent did not correlate with the levels of depression, anxiety, or stress.

**Table 6:** Correlations between depression, anxiety and stress levels and different variables

Variables	Depression	***p***-value	Anxiety	***p***-value	Stress	***p***-value
Age	−0.350**	0.000	−0.190**	0.000	−0.200**	0.000
Number of children	−0.288**	0.000	−0.145**	0.000	−0.162**	0.000
Subjective perception of overall health	−0.224**	0.000	−0.274**	0.000	−0.252**	0.000
Subjective perception of mental health	−0.597**	0.000	−0.479**	0.000	−0.561**	0.000
Concern regarding contracting COVID-19	0.122**	0.002	0.244**	0.000	0.244**	0.000
Number of cohabitants	−0.011	0.778	0.031	0.438	−0.015	0.709

** Correlation is significant at 0.01 level l (2-tailed)

### Univariate and multivariate ordinal logistic regression analysis for depression, anxiety, and stress

As shown in Table 7, univariate ordinal regression analyses demonstrated that being male (OR = 0.66; 95% CI = 0.46–0.93, *p:* 0.02), being  older (OR = 0.92; 95% CI = 0.90–0.94, *p:* 0.0001), and having more children (OR = 0.45; 95% CI = 0.35–0.58, *p:* 0.0001) were all associated with reduced odds of having more severe levels of depression. On the other hand, being a student (OR = 3.67; 95% CI = 2.56–5.26, *p:* 0.0001) and having a relative diagnosed with COVID-19 (OR= 1.70; 95% CI= 1.03–2.80, *p:* 0.036) were associated with relatively higher odds of having more severe levels of depression. All of the statistically significant variables in the univariate analysis were included in the multivariate ordinal regression analysis. In this model, only male sex (OR = 0.64; 95% CI = 0.44–0.93, *p:* 0.019),  older age (OR = 0.96; 95% CI = 0.92–0.99, *p:* 0.027), and more children (OR = 0.71; 95% CI = 0.50–0.99, *p:* 0.044) remained significantly associated with reduced odds of having more severe levels of depression.

**Table 7:** Univariate and multivariate ordinal regression analyses of sociodemographic characteristics associated with depression, anxiety and stress

Variables	Univariate			Multivariate		
	OR	95% CI	***p-***value	OR	95% CI	***p-***value
**Depression**						
Male sex	0.66	0.46–0.93	0.02	0.64	0.44–0.93	0.019
Age	0.92	0.90–0.94	0.0001	0.96	0.92–0.99	0.027
Location	1.14	0.80–1.62	0.487			
Number of children	0.45	0.35–0.58	0.0001	0.71	0.50–0.99	0.044
Number cohabitants	0.99	0.90–1.09	0.963			
Student status	3.67	2.56–5.26	0.0001	1.51	0.92–2.48	0.097
Cohabitants with physical and/or intellectual disabilities	1.11	0.66–1.89	0.681			
COVID-19 symptoms	1.34	0.87–2.05	0.172			
COVID-19 diagnosis	0.95	0.46–1.97	0.899			
Relative diagnosed with COVID-19	1.70	1.03–2.80	0.036	1.64	0.97–2.77	0.060
**Anxiety**						
Male sex	0.48	0.34–0.67	0.0001	0.45	0.32–0.63	0.0001
Age	0.96	0.95–0.98	0.0001	0.97	0.95–1.00	0.082
Location	1.06	0.76–1.47	0.7			
Number of children	0.78	0.67–0.9	0.001	0.96	0.76–1.21	0.75
Number of cohabitants	1.04	0.95–1.13	0.35			
Student status	1.86	1.35–2.55	0.0001	1.23	0.78–1.92	0.35
Cohabitants with physical and/or intellectual disabilities	1.05	0.63–1.72	0.84			
COVID-19 symptoms	2.23	1.51–3.31	0.0001	2.07	1.33–3.22	0.001
COVID-19 diagnosis	1.82	0.96–3.42	0.06			
Relative diagnosed with COVID-19	2.17	1.35–3.47	0.001	1.42	0.84–2.40	0.18
**Stress**						
Male sex	0.39	0.26–0.60	0.0001	0.38	0.25–0.59	0.0001
Age	0.95	0.93–0.97	0.0001	0.96	0.93–1.00	0.085
Location	1.08	0.73–1.60	0.68			
Number of children	0.64	0.51–0.80	0.0001	0.83	0.60–1.15	0.276
Number of cohabitants	1.00	0.90–1.11	0.989			
Student status	2.17	1.47–3.19	0.0001	1.10	0.64–1.89	0.718
Cohabitants with physical and/or intellectual disabilities	1.22	0.68–2.17	0.496			
COVID-19 symptoms	1.49	0.94–2.37	0.087			
COVID-19 diagnosis	1.34	0.63–2.84	0.442			
Relative diagnosed with COVID-19	1.69	0.98–2.91	0.056			

Intriguingly, being male (OR = 0.48; 95% CI = 0.34–0.67, *p:* 0.0001), being older (OR = 0.96; 95% CI = 0.95–0.98, *p:* 0.0001), and having more children (OR = 0.78; 95% CI = 0.67–0.9, *p:* 0.001) were also associated with reduced odds of having more severe levels of anxiety. Concurrently, other variables such as being a student (OR= 1.86; 95% CI= 1.35–2.55, *p:* 0.0001), having COVID-19 symptoms (OR = 2.23; 95% CI= 1.51–3.31, *p:* 0.0001), and having a relative diagnosed with COVID-19 (OR = 2.17; 95% CI= 1.35–3.47, *p:* 0.001) were all associated with relatively higher odds of having more severe levels of anxiety. In the multivariate analysis, male sex (OR = 0.45; 95% CI = 0.32–0.63 *p:* 0.0001) was the only variable that remained statistically significantly associated with reduced odds of having more severe levels of anxiety, whereas having COVID-19 symptoms (OR = 2.07; 95% CI = 1.33–3.22, *p:* 0.001) was the only variable that remained statistically significantly associated with relatively higher odds.

Finally, in the stress models, male sex (OR = 0.39; 95% CI = 0.26–0.60, *p:* 0.0001), older age (OR = 0.95; 95% CI = 0.93–0.97, *p:* 0.0001), and more children (OR = 0.64; 95% CI = 0.51–0.80, *p:* 0.0001) were associated with reduced odds of having more severe levels of stress. However, being a student (OR = 2.17; 95% CI = 1.47–3.19, *p:* 0.0001) was associated with relatively higher odds of having more severe levels of stress. Finally, the multivariate analysis showed that male sex (OR = 0.39; 95% CI = 0.25–0.60, *p:* 0.0001) was the only variable statistically significantly associated with reduced odds of having more severe levels of stress.

## Discussion

This study assessed the levels of depression, anxiety and stress among the general population in Ecuador during social isolation due to the COVID-19 outbreak. Our results showed that the most common mental health issue was anxiety, with 30.7% of the respondents reporting moderate to very severe anxiety levels, followed by depression (17.7%) and stress (14.2%). Similar findings were reported by a Chinese study in which the most common mental health issue was also moderate to severe anxiety (28.8%), followed by depression (16.5%) and stress (8.1%) [18]. In contrast, a Spanish study revealed that the most prevalent issue was depression (29.6%), rather than anxiety (25.3%) or stress (22.4%) [19]. This analysis contributes to our understanding of the behavior of the Ecuadorian population in comparison with that of their communities [14, 20–22].

Our study determined that women have significantly higher levels of depression, anxiety, and stress than males, which tends to be a common finding in most studies around the world [10, 18, 19, 23]. Restrictive measures regarding schools and daycare centers may significantly increase the burden on women at home, leading to fatigue and a reduction in their work performance [11, 19, 24]. On the other hand, an increase in domestic violence against women during quarantine due to the pandemic and a higher risk of losing their job and income could be the reasons for our findings [24–28].

Verma and Mishra reported that male sex is associated with reduced odds of stress; however, their findings also suggest that being male is associated with greater odds of depression and anxiety [29]. In contrast, our study determined that being male is associated with reduced odds of having severe levels of depression, anxiety, and stress. Studies in Latin America have shown that men have significantly lower levels of depression, stress and anxiety than women [14, 15, 20].

A recent systematic review and meta-analysis stated that younger adults (21–40 years) constituted the most severely affected population [18, 21], with a high risk of mental health issues during the COVID-19 pandemic [8, 19, 20, 23, 30]. In fact, we found inverse correlations between age and the levels of depression, anxiety, and stress. The reason may be that younger individuals tend to be more concerned about future consequences and the negative impact of the pandemic on the global economy and job availability [18, 21]. Similarly, young people have greater and more continuous access to worrisome and/or inaccurate information due to their use of social media, which can affect their mental health [10, 25, 26, 31, 32]. Additionally, young people tend to be students, for whom uncertainty and the lack of academic progression are sources of significant distress [18]. In our study, students had significantly higher levels of depression, anxiety, and stress, which is a common finding of other studies [18, 19, 23].

The relationship status of the respondents seemed to influence the severity of mental health issues. We found that married people tended to have significantly lower levels of depression, anxiety, and stress than single people, as reported in other studies [19, 23, 33]. The number of children also appeared to have an effect on the psychological impact of social isolation. Our study showed inverse correlations between the number of children and the levels of depression, anxiety and stress. The more children an individual had, the lower the level of mental distress. Similarly, other studies have also found that having a child was associated with lower levels on each subscale of the DASS-21 [9, 34–36]. Interestingly, the number of cohabitants was not correlated with the levels of depression, anxiety, and depression, unlike the Spanish population, where a household with 2 people was likely to experience a reduced psychological impact [19].

A more favorable perception of health was associated with a reduced psychological impact [9, 19]. Similarly, we found that a higher perception of overall health was negatively correlated with the levels of depression, anxiety, and stress. Having a variety of symptoms compatible with COVID-19 was associated with a significant increase in the odds of more severe levels of anxiety, as reported in previous studies [11, 18, 19]. However, having a positive COVID-19 diagnosis was not associated with more anxiety in our population. This finding seems paradoxical because research has shown that COVID-19 patients experience significant psychological distress [37]. A possible reason may be that most of these patients were not hospitalized; therefore, they were probably experiencing a mild case of COVID-19. A recent Ecuadorian study assessed depression and anxiety in patients with confirmed or suspected COVID-19 diagnoses using the Generalized Anxiety Disorder 7-item (GAD-7) and Patient Health Questionnaire-9 (PHQ-9) questionnaires. Those findings suggested that the severity of depressive symptoms was significantly greater in the group with confirmed COVID-19, although there was no effect on the severity of anxiety symptoms. One of the major limitations of that study was that the researchers could not determine if the presence of psychological symptoms was the result of being under surveillance or the disease itself. Therefore, the researchers recognized the need for a study focusing on the general population [38].

On the other hand, having a relative diagnosed with COVID-19 was associated with more severe levels of depression and anxiety. This result is similar to some findings from China, where people were more worried about their relatives contracting COVID-19 but were less distressed when they themselves were infected, remaining optimistic that they would survive the disease [18]. Based on these findings, we believed that subjective perceptions (e.g., overall health, mental health, and COVID-19 compatible symptoms) were more strongly correlated with a negative psychological impact than more objective measurements, such as a confirmed COVID-19 diagnosis. A possible explanation is that isolated people perceived themselves as being more vulnerable than they actually were [19].

Furthermore, our research suggested that the COVID-19 pandemic, along with social isolation measures, has definitely affected the mental health of the general population in our country. Hopefully, some interventions can be initiated to improve the mental health of Ecuadorians during the pandemic. The first step should be to raise awareness of the current mental health issues due to the COVID-19 pandemic to provide information and guidelines to help identify individuals in need of appropriate help.

Online psychological assistance and telephone counseling should be provided to address this issue [39]. In addition, cognitive behavioral therapy (CBT) does not always require the assistance of a mental health professional [18], which makes this a cost-effective intervention. Our study identified high-risk groups, such as females, students, young adults, unmarried people, and individuals with COVID-19 symptoms, who were found to be more vulnerable to greater psychological impact. Based on this, we recommend that educational institutions and workplaces arrange psychological tests to determine the mental health status of their students and workers, especially females, thereby identifying who is in need of further psychological support.

One of the main strengths of this study was that it included a relatively large sample. However, with regard to the interpretation of our findings, there are several limitations worth mentioning. First, we adopted the snowball sampling method due to limitations with regard to time and resources. This prevented our study population from being randomly selected and led to an oversampling of participants from a particular region (Guayas/Guayaquil). Additionally, young individuals accounted for a significant proportion of our sample, which may be due to the use of social media as the primary broadcast channel for the survey. Furthermore, our results could be different in other Latin American countries, limiting the generalizability of our results. Another major limitation was that the levels of depression, anxiety, and stress were self-reported by the respondents. Finally, due to the large number of asymptomatic patients and the lack of widespread COVID-19 testing in the early stages of the pandemic, it is possible that a considerable number of the respondents had been infected with SARS-CoV-2 but were unaware of that fact at the time of the survey. Nevertheless, our findings provide valuable information about mental health in a Latin American country during the social isolation period of the current COVID-19 pandemic.

## Conclusion

Social isolation measures in Ecuador during the COVID-19 pandemic have negatively impacted individuals and disrupted their lives. Approximately one-third of the sample population had mental health problems, with females, younger individuals and students experiencing more severe negative psychological impacts. The design and early implementation of tailored strategies and physiological interventions could help avoid more severe problems. More research will be needed to evaluate these early interventions and the long-term benefits.

## Supplementary Information

**Additional file 1.**

## Data Availability

All data generated or analyzed during this study are included in this published article (as a supplementary file in SPSS format).

## Abbreviations

COVID-19: Coronavirus disease 2019
SARS-CoV-2: Severe acute respiratory syndrome coronavirus
WHO: World Health Organization
PTSD: Posttraumatic stress disorder
DASS-21: Depression, Anxiety and Stress Scale-21 Items
GAD-7: Generalized Anxiety Disorder 7-items
PHQ-9: Patient Health Questionnaire-9
CBT: Cognitive behavioral therapy
